# Supplementary material for: The genetic factors of bilaterian evolution
Source: eLife. 2020 Jul 16;9:e45530. doi: 10.7554/eLife.45530 (PMC7535936; doi:10.7554/eLife.45530)
Supplement: Supplementary file 1. [file elife-45530-supp1.pdf]

The genetic factors of bilaterian evolution.

Peter Heger, Wen Zheng, Anna Rottmann, Kristen A. Panfilio, Thomas Wiehe

Supporting Information

Species	Read Archive ID	CEGMA [%]	Web address for download
Aiptasia pallida	?	41.1	https://www.ncbi.nlm.nih.gov/sra/?term=SRX202151 & SRX231866
Anemonia viridis	LIBEST_023433	15.7	https://www.ncbi.nlm.nih.gov/biosample/?term=LIBEST_023433
Beroe sp.	ERR216194	27.8	https://trace.ncbi.nlm.nih.gov/Traces/sra/?run=ERR216194
Brachionus calyciflorus	?	92.7	https://trace.ncbi.nlm.nih.gov/Traces/sra/?run=SRR611718 & SRR611719,SRR611720
Brachionus calyciflorus5.6	?	–	https://www.ncbi.nlm.nih.gov/sra/SRX203183[accn]
Bugula neritina	SRR034781	14.5	https://trace.ncbi.nlm.nih.gov/Traces/sra/?run=SRR034781
Cephalothrix hongkongiensis	SRR618505	75.0	https://trace.ncbi.nlm.nih.gov/Traces/sra/?run=SRR618505
Chiton olivaceus	SRR618506	55.2	https://trace.ncbi.nlm.nih.gov/Traces/sra/?run=SRR618506
Eupolybothrus cavernicolus	ERR338470	85.1	https://trace.ncbi.nlm.nih.gov/Traces/sra/?run=ERR338470
Evechinus chloroticus	?	91.1	https://trace.ncbi.nlm.nih.gov/Traces/sra/?run=SRR1014618 & SRR1014619
Hermodice carunculata	SRR651044	93.9	https://trace.ncbi.nlm.nih.gov/Traces/sra/?run=SRR651044
Hormogaster elisae	?	97.2	https://trace.ncbi.nlm.nih.gov/Traces/sra/?run=SRR786597 & SRR786598
Lingula anatina	SRR330440	8.1	https://trace.ncbi.nlm.nih.gov/Traces/sra/?run=SRR330440
Metasiro americanus	SRR618563	92.7	https://trace.ncbi.nlm.nih.gov/Traces/sra/?run=SRR618563
Mnemiopsis leidyi	LIBEST_014587	–	https://www.ncbi.nlm.nih.gov/nucest/?term=LIBEST_014587
Mnemiopsis leidyi	LIBEST_022347	21.8	https://www.ncbi.nlm.nih.gov/nucest/?term=LIBEST_022347
Mnemiopsis leidyi	?	–	https://research.nhgri.nih.gov/mnemiopsis/download/proteome/ML2.2.aa.gz
Myxobolus cerebralis	SRR628302	26.6	https://trace.ncbi.nlm.nih.gov/Traces/sra/?run=SRR628302
Nasoonaria sinensis	SRR1048661	86.7	https://trace.ncbi.nlm.nih.gov/Traces/sra/?run=SRR1048661
Patiria miniata	SRR053628	–	https://trace.ncbi.nlm.nih.gov/Traces/sra/?run=SRR053628
Patiria miniata	SRR1138705	–	https://trace.ncbi.nlm.nih.gov/Traces/sra/?run=SRR1138705
Patiria miniata	SRX021529	96.0	https://www.ncbi.nlm.nih.gov/sra/SRX021529[accn]
Patiria miniata	SRX021531	–	https://www.ncbi.nlm.nih.gov/sra/SRX021531[accn]
Ptychodera flava	SRR1029584	55.6	https://trace.ncbi.nlm.nih.gov/Traces/sra/?run=SRR1029584
Urticina eques	SRR942796	11.7	https://trace.ncbi.nlm.nih.gov/Traces/sra/?run=SRR942796

Supplementary Table 1: **Transcriptome sequence download links and CEGMA results for the species used in BigWenDB.** The left column indicates the species from which we downloaded transcriptomes and generated transcriptomic ORFs. The respective download address is shown in the right column.

Species	Taxon ID	Phylum	CEGMA score
Anemonia viridis	51769	Cnidaria	16 %
Acropora cervicornis	6130	Cnidaria	79.44 %
Acropora millepora	45264	Cnidaria	51.61 %
Aiptasia pallida	12923	Cnidaria	41 %
Aphrocallistes vastus	83887	Porifera	94.35 %
Beroe abyssicola	320166	Ctenophora	84.27 %
Bolocera tuediae	1163374	Cnidaria	32 %
Beroe sp.	220132	Ctenophora	28 %
Crella elegans	252961	Porifera	22.18 %
Gorgonia ventalina	204384	Cnidaria	93 %
Hydractinia symbiolongicarpus	13093	Cnidaria	71.77 %
Myxobolus cerebralis	59783	Cnidaria	26 %
Nanomia bijuga	168759	Cnidaria	87 %
Hormathia digitata	1163372	Cnidaria	45 %
Montastraea faveolata	48498	Cnidaria	25.4 %
Metridium senile	6116	Cnidaria	16.13 %
Oscarella carmela	386100	Porifera	99 %
Petrosia ficiformis	68564	Porifera	94 %
Porites australiensis	51061	Cnidaria	95.97 %
Stylophora pistillata	50429	Cnidaria	67.34 %
Suberites domuncula	55567	Porifera	20.97 %
Bolinopsis infundibulum	140455	Ctenophora	69.35 %
Euplokamis dunlapae	1403701	Ctenophora	56.45 %
Urticina eques	417072	Cnidaria	11.69 %
Vallicula multiformis	140489	Ctenophora	66.53 %

Supplementary Table 2: CEGMA results for 25 transcriptomes of non-bilaterian metazoans. The BigWenDB contains sequence data from 33 non-bilaterian metazoans. For eight non-bilaterian species, including four cnidarians, we collected genomic ORF data (Figure 1–Figure Supplement 1), they are not listed here. CEGMA results of 15 additional cnidarian species are highlighted in red. Each of the non-bilaterian phyla—Porifera, Ctenophora, and Cnidaria—is covered by several species with high CEGMA score.

Species	Superphylum	Web address for download
Acropora digitifera	nB	https://www.ncbi.nlm.nih.gov/nuccore/BACK00000000
Alatina moseri	nB	https://www.ncbi.nlm.nih.gov/nuccore/AHZO00000000
Amphimedon queenslandica	nB	https://www.ncbi.nlm.nih.gov/nuccore/ACUQ01000000
Caenorhabditis elegans	E	https://www.ncbi.nlm.nih.gov/assembly/GCA_000002985.3#/def
Callorhinchus milii	D	https://www.ncbi.nlm.nih.gov/nuccore/AAVX02000000
Capitella teleta	L	https://www.ncbi.nlm.nih.gov/nuccore/AMQN01000000
Crassostrea gigas	L	https://www.ncbi.nlm.nih.gov/nuccore/AFTI01000000
Drosophila melanogaster	E	https://www.ncbi.nlm.nih.gov/nuccore/AABU00000000
Helobdella robusta	L	https://www.ncbi.nlm.nih.gov/nuccore/AMQM01000000
Homo sapiens	D	https://www.ncbi.nlm.nih.gov/assembly/GCA_000001405.26
Hydra magnipapillata	nB	https://www.ncbi.nlm.nih.gov/nuccore/ACZU01000000
Latimeria chalumnae	D	https://www.ncbi.nlm.nih.gov/nuccore/BAHO01000000
Lottia gigantea	L	https://www.ncbi.nlm.nih.gov/nuccore/AMQO01000000
Mnemiopsis leidyi	nB	https://www.ncbi.nlm.nih.gov/nuccore/AGCP01000000
Nematostella vectensis	nB	https://www.ncbi.nlm.nih.gov/nuccore/ABAV01000000
Parasteatoda tepidariorum	E	https://www.ncbi.nlm.nih.gov/nuccore/AOMJ00000000
Pleurobrachia bachei	nB	https://www.ncbi.nlm.nih.gov/nuccore/AVPN01000000
Priapulus caudatus	E	https://www.ncbi.nlm.nih.gov/nuccore/AXZU00000000
Romanomermis culicivorax	E	https://www.ncbi.nlm.nih.gov/nuccore/CAQS00000000
Saccoglossus kowalevskii	D	https://www.ncbi.nlm.nih.gov/nuccore/ACQM01000000
Schistosoma japonicum	L	https://www.ncbi.nlm.nih.gov/nuccore/CABF01000000
Strigamia maritima	E	https://www.ncbi.nlm.nih.gov/nuccore/AFFK01000000
Strongylocentrotus purpuratus	D	https://www.ncbi.nlm.nih.gov/nuccore/AAGJ00000000
Tribolium castaneum	E	https://www.ncbi.nlm.nih.gov/nuccore/AAJJ00000000
Trichoplax adhaerens	nB	https://www.ncbi.nlm.nih.gov/nuccore/ABGP01000000

Supplementary Table 3: Genome sequence download links for 25 species. The left column indicates 25 species for which we downloaded genome assemblies and generated genomic ORFs (download address see right column). Their assignment to one of four superphyla is denoted in the central column. nB = non-Bilateria; D = Deuterostomia; E = Ecdysozoa; L = Lophotrochozoa.

Command	Output of OrthoMCL, original version and R version		
`wc -l BLAST_output_tbl`	243,586,115		
`wc -l orthologs.txt`	56,298		
`md5sum orthologs.txt`	bb1f573a1a655bc1823b0cedd15e323f		
`head -5 orthologs.txt`	Query	Subject	Score
	283909|orf_AMQN01000004.1_7_0	400682|orf_ACUQ01027136.1_5_0	1.013
	283909|orf_AMQN01000004.1_9_0	400682|orf_ACUQ01002585.1_372_0	0.731
	283909|orf_AMQN01000013.1_352_0	400682|orf_ACUQ01002623.1_320_0	0.34
	283909|orf_AMQN01000022.1_395_0	400682|orf_ACUQ01000660.1_549_0	0.578
	283909|orf_AMQN01000022.1_399_0	400682|orf_ACUQ01000660.1_553_0	0.31
`wc -l inparalogs.txt`	36,169,614		
`md5sum inparalogs.txt`	f7022d81bdbac55fa3bdd0ce5f43ab1d		
`head -5 inparalogs.txt`	Query	Subject	Score
	283909|orf_AMQN01000002.1_10_0	283909|orf_AMQN01009125.1_12_0	0.464
	283909|orf_AMQN01000002.1_198_0	283909|orf_AMQN01000122.1_693_0	0.247
	283909|orf_AMQN01000002.1_198_0	283909|orf_AMQN01000957.1_1014_0	0.184
	283909|orf_AMQN01000002.1_198_0	283909|orf_AMQN01001209.1_66_0	0.238
	283909|orf_AMQN01000002.1_198_0	283909|orf_AMQN01003462.1_223_0	0.157
`wc -l coorthologs.txt`	17,118		
`md5sum coorthologs.txt`	ea58aaa7e148d5bb6182e821c360e655		
`head -5 coorthologs.txt`	Query	Subject	Score
	283909|orf_AMQN01000013.1_352_0	400682|orf_ACUQ01002623.1_295_0	0.178
	283909|orf_AMQN01000051.1_3573_0	400682|orf_ACUQ01007187.1_362_0	0.349
	283909|orf_AMQN01000059.1_392_0	400682|orf_ACUQ01018084.1_12_0	0.249
	283909|orf_AMQN01000061.1_168_0	400682|orf_ACUQ01004990.1_369_0	0.585
	283909|orf_AMQN01000061.1_168_0	400682|orf_ACUQ01004991.1_413_0	0.585

Supplementary Table 4: Comparison between the standard OrthoMCL pipeline and our R version. To create a test dataset for the R version of OrthoMCL, we merged the first 100 (of 10,000) BLAST output tables of the full dataset, comprising 243,586,115 BLAST hits, and processed the resulting file in parallel with the R version of OrthoMCL and with the original version [Li et al. 2003]. Both pipelines delivered identical ortholog, inparalog, and coortholog tables which serve as input for the final clustering steps. This table shows the number of rows present in the BLAST output table (`wc -l BLAST_output_tbl`) and in the three OrthoMCL output tables (`wc -l orthologs.txt inparalogs.txt coorthologs.txt`) as well as the associated MD5 message digest checksums. In addition, the first five rows of all three orthology tables are displayed (`head -5 orthologs.txt inparalogs.txt coorthologs.txt`). All numbers and row contents are identical between the original and the R version of OrthoMCL.

**Number of . . .**	**Three tables** (incl. inparalog table)	**Two tables** (excl. inparalog table)
collected sequences	124,031,501	124,031,501
seq in blast table	122,149,508	122,149,508
blast pairs in total	5,966,491,524	5,966,491,524
seq pairs in ortholog table	92,901,096	92,901,096
seq pairs in co-ortholog table	25,598,428	25,598,428
seq pairs in in-paralog table	688,344,999	—
seq pairs in total	806,844,523	118,499,524
Orthologous groups (OGs)	4,049,532	824,605
ORF seq in OGs	28,211,771	2,387,557
TRS seq in OGs	4,633,170	2,207,632
NCBI seq in OGs	2,210,531	2,032,530
seq in OGs in total	35,227,294	6,743,519
seq not in OGs	86,922,214	115,405,989
OGs with $\leq$3 species	3,854,629	637,895
OGs with $\geq$10 species	74,398	75,744
OGs with $\leq$3 seq	74.8 %: 3,028,635	71.8 %: 592,260
OGs with $\geq$10 seq	365,492	97,967

Supplementary Table 5: Summary statistics of Markov clusterings. The central column ("Three tables") displays basic clustering results from using all three orthology tables (ortholog, co-ortholog, and inparalog table), while the right column reports results obtained from clustering with the ortholog and co-ortholog tables. For all further analyses, the two-table results without inparalog table were used.

Ortholog *Drosophila*		Ortholog *Homo*		BigWen OG	Composition of OG				
					Po	Pl	Ct	Cn	Bi
tin	[AAF55890]	NKX2.3<br>NKX2.5<br>NKX2.6	[NP_660328]<br>[NP_004378]<br>[A6NCS4]	OG_92160 (Dm)<br>OG_613 (Hs)	0<br>1	0<br>**1**	0<br>2	0<br>16	10<br>128
bap	[AAF55891]	NKX3.1<br>NKX3.2	[Q99801]<br>[P78367]	OG_7136	4	0	0	8	94
lbe*<br>lbl	[CAA70056]<br>[NP_001262805]	LBX1<br>LBX2	[P52954]<br>[Q6XYB7]	OG_8094	1	0	2	9	85
C15	[AAF55898]	TLX1<br>TLX2<br>TLX3	[P31314]<br>[O43763]<br>[O43711]	OG_7799	3	0	6	2	81
slou	[P22807]	NKX1.1<br>NKX1.2	[Q9UD57]<br>[NP_001139812]	OG_7615	1	0	1	7	91

Supplementary Table 6: Orthogroup composition of NK cluster genes in the BigWen database. The table shows, from left to right, *Drosophila* NK genes, their corresponding human orthologs and paralogs (GenBank accession numbers in square brackets), the orthogroup(s) to which an NK gene was assigned in our dataset, and the composition of this orthogroup, denoting the number of species from Po (Porifera), Pl (Placozoa), Ct (Ctenophora), Cn (Cnidaria), and Bi (Bilateria) that belong to this group. In contrast to other NK genes, *D. melanogaster* tinman is not placed with its human counterparts, but is a member of an extra orthogroup restricted to Endopterygota (OG_92160, see text). NKX2.3 is the only NK cluster gene with a potential ortholog in Placozoa (bold). *: Lbe/lbl in *Drosophila* and LBX1/LBX2 in *Homo* are both independent duplications of a single ancestral ladybird gene [Cande et al. 2009] and not orthologous to each other. Left column: tin (tinman), bap (bagpipe), lbe (ladybird early), lbl (ladybird late), slou (slouch).

OG	Name	OG comp.	HMM hits	OG comp.	Ancestor	E-value
OG_7767	Sprouty	1 Pl; 85 B	1. OG_46647	15 Cn; 2 L	Eumetazoa	$6.0e^{-30}$
			2. OG_58139	13 Cn; 1 E	Eumetazoa	$1.8e^{-27}$
			3. OG_73898	8 Cn; 1 E	Eumetazoa	$2.1e^{-23}$
OG_46647	Sprouty-2	15 Cn; 2 L	1. OG_58139	13 Cn; 1 E	Eumetazoa	$6.2e^{-36}$
			2. OG_7767	1 Pl; 85 B	Metazoa	$1.3e^{-29}$
			3. OG_73898	8 Cn; 1 E	Eumetazoa	$7.4e^{-27}$
OG_58139	Sprouty-3	13 Cn; 1 E	1. OG_46647	15 Cn; 2 L	Eumetazoa	$1.7e^{-36}$
			2. OG_7767	1 Pl; 85 B	Metazoa	$2.9e^{-27}$
			3. OG_73898	8 Cn; 1 E	Eumetazoa	$2.9e^{-25}$
OG_73898	Sprouty-4	8 Cn; 1 E	1. OG_46647	15 Cn; 2 L	Eumetazoa	$3.4e^{-27}$
			2. OG_58139	13 Cn; 1 E	Eumetazoa	$2.3e^{-25}$
			3. OG_7767	1 Pl; 85 B	Metazoa	$6.4e^{-23}$
OG_56080	GAF	13 Diptera	1. OG_165721	5 Pterygota	Pterygota	$1.9e^{-73}$
			2. OG_405835	2 Sophophora	Sophophora	$5.9e^{-52}$
			3. OG_26633	23 Pancrustacea	Pancrustacea	$4.8e^{-28}$
OG_165721	GAF-2	5 Pterygota	1. OG_56080	13 Diptera	Diptera	$4.2e^{-71}$
			2. OG_26633	23 Pancrustacea	Pancrustacea	$1.2e^{-26}$
			3. OG_34593	21 Hexapoda	Hexapoda	$2.8e^{-26}$
OG_26633	n.d.	23 Pancrustacea	1. OG_34593	21 Hexapoda	Hexapoda	$7.6e^{-58}$
			2. OG_6341	1 Cn; 2 Ct; 37 E; 1 L	Metazoa	$1.1e^{-57}$
			3. OG_157127	5 Endopterygota	Endopterygota	$2.7e^{-56}$

Supplementary Table 7: The HMM-HMM reciprocal best hit strategy for orthogroup completion. Orthogroup ID, corresponding gene name, and orthogroup composition is shown for several reciprocal HMM-HMM best hit orthogroups of two example proteins, Sprouty and GAGA factor. Columns 4–7 summarise results of HMM-HMM searches in our database. In column four and five, orthogroup ID and composition of the three best search hits for each query is shown. Column six and seven report last common ancestor and corresponding E-value of hit orthogroups. B = Bilateria; Cn = Cnidaria; Ct = Ctenophora; D = Deuterostomia; E = Ecdysozoa; L = Lophotrochozoa; Pl = Placozoa; n.d. = not determined. Orthogroups are coloured to illustrate reciprocal best hit relationships. Note that the four orthogroups OG_7767, OG_46647, OG_58139, and OG_73898 are each other's hits in reciprocal HMM-HMM searches, suggesting that a combination of the four orthogroups with the inferred ancestor "Metazoa" (ancestor of bilaterians, cnidarians, and placozoans) correctly reflects evolutionary history, in agreement with the description of Sprouty in cnidarians [Matus et al. 2007]. Similarly, OG_56080 and OG_165721 are each other's reciprocal best hit while another orthogroup, OG_26633, does not satisfy this criterion. The complete orthogroup for GAGA factor is therefore a combination of OG_56080 and OG_165721 with the ancestor "Pterygota", as published previously [Heger et al. 2013].

OG	Uniprot ID Hs	# ZF	Gene name Dm	# ZF
OG_5117	BC11A_HUMAN	6	CG9650 (Q494J4_DROME)	5
OG_5533	CASZ1_HUMAN	8	castor (CAS_DROME)	4
OG_5543	EVI1_HUMAN	10	CG10348 (Q4V722_DROME)	3
OG_6452	CTCF_HUMAN	11	CTCF (Q9VS55_DROME)	11
OG_6894	ZN384_HUMAN	8	rotund (RN_DROME)	6
OG_7047	ZN236_HUMAN	30	XP_001807307 (*T. castaneum*)	23
OG_8049	ZN521_HUMAN	30	O/E-ass. zinc finger protein (A0A0B4JD76_DROME)	9
OG_8738	ZN407_HUMAN	22	CG31612 (Q9V9Q2_DROME)	7
OG_9930	PRD10_HUMAN	10	XP_974598 (*T. castaneum*)	13
OG_10418	ZFAT_HUMAN	19	126957|predict_SMAR002996-PA_0 (*S. maritima*)	18
OG_11041	PLAL1_HUMAN	7	XP_975272 (*T. castaneum*)	7
OG_11354	10224|gi_297139771	5	schnurri (Q24605_DROME)	8
OG_14415	ZF64B_HUMAN	13	126957|predict_SMAR005066-PA_0 (*S. maritima*)	7

Supplementary Table 8: Bilaterian-specific poly-zinc finger transcription factors. First column: Orthogroup ID of 13 bilaterian-specific zinc finger proteins with tandem C_2H_2 domains (according to BigWenDB). Second and third column: Uniprot ID and number of zinc finger domains in the human ortholog; for OG_11354, the *Saccoglossus kowalevskii* ortholog is listed because no chordate species are present in the group. Fourth column: Name and Uniprot ID (in parentheses) of the *D. melanogaster* ortholog. In orthogroups without *Drosophila* sequence, the corresponding *T. castaneum* or *S. maritima* name and ID is listed. Fifth column: Number of zinc finger domains of the corresponding arthropod ortholog. *T. castaneum*: *Tribolium castaneum* (Insecta), *S. maritima*: *Strigamia maritima* (Myriapoda).

OG	Best hit in BigWenDB	E-value	Best hit in non-Bilateria	E-value
OG_8220	283909|gi_443712155	$1.9e^{-90}$	68564|trs_comp42343_c0_seq14_4_0	$6.2e^{-10}$
OG_13336	7868|predict_SINCAMP*	$3.0e^{-71}$	45351|gi_156229131	$2.0e^{-05}$
OG_28197	8478|gi_530579811	$4.4e^{-60}$	68564|trs_comp25246_c0_seq1_8_0	$2.3e^{-12}$
OG_31055	137513|trs_comp59583*	$9.9e^{-31}$	204384|trs_comp110987_c0_seq3_7_0	$4.5e^{-23}$
OG_33174	10090|gi_148690596	$5.4e^{-43}$	68564|trs_comp43046_c0_seq89_19_0	$2.2e^{-09}$

Supplementary Table 9: Novel protein domains of bilaterian origin. Left column: Orthogroup identifier in BigWenDB. Second and third column: Sequence ID and E-value of the best HMM search hit in the entire BigWenDB (HMM search in fasta-formatted BigWenDB sequence collection). In contrast, the fourth and last column show sequence ID and E-value of the best HMM search hit from non-bilaterian metazoans. *: abbreviated for space constraints. Full ID is 7868|predict_SINCAMP00000020970_0 and 137513|trs_comp59583_c0_seq3_48_0, respectively. Red colour indicates the only bilaterian-specific orthogroup for which we found similar sequences of non-bilaterian origin, indicating the existence of non-orthologous proteins with a similar domain in non-bilaterians metazoans.

OG	Name	Function
		Homeobox domain
4203	Homeobox protein Hox-D8	Sequence-specific transcription factor which is part of a developmental regulatory system that provides cells with specific positional identities on the anterior-posterior axis.
5339	Prospero homeobox protein 2	Transcription factor involved in developmental processes such as cell fate determination, gene transcriptional regulation and progenitor cell regulation in a number of organs. Plays a critical role in embryonic development and functions as a key regulatory protein in neurogenesis and the development of the heart, eye lens, liver, pancreas and the lymphatic system. Involved in the regulation of the circadian rhythm. [...]
11804	Homeobox-containing protein 1	Transcription factor. Isoform 1 acts as a transcriptional repressor. Isoform 4 has very low activity as a transcriptional repressor.
11810	Paired mesoderm homeobox protein 1	Acts as a transcriptional regulator of muscle creatine kinase (MCK) and so has a role in the establishment of diverse mesodermal muscle types. The protein binds to an A/T-rich element in the muscle creatine enhancer.
12894	Homeobox protein Mohawk	May act as a morphogenetic regulator of cell adhesion.
18424	Intestine-specific homeobox	Transcription factor that regulates gene expression in intestine. May participate in vitamin A metabolism most likely by regulating BCO1 expression in the intestine.
		bHLH domain
5116	Class E basic helix-loop-helix protein 22	Inhibits DNA binding of TCF3/E47 homodimers and TCF3 (E47)/NEUROD1 heterodimers and acts as a strong repressor of Neurod1 and Myod-responsive genes, probably by heterodimerization with class a basic helix-loop-helix factors. Despite the presence of an intact basic domain, does not bind to DNA (By similarity). In the brain, may function as an area-specific transcription factor that regulates the postmitotic acquisition of area identities and elucidate the genetic hierarchy between progenitors and postmitotic neurons driving neocortical arealization. [...]
6893	Myoblast determination protein 1	Acts as a transcriptional activator that promotes transcription of muscle-specific target genes and plays a role in muscle differentiation. Together with MYF5 and MYOG, co-occupies muscle-specific gene promoter core region during myogenesis. Induces fibroblasts to differentiate into myoblasts. Interacts with and is inhibited by the twist protein. This interaction probably involves the basic domains of both proteins. [...]
7866	Neuronal PAS domain-containing protein 4	Transcription factor expressed in neurons of the brain that regulates the excitatory-inhibitory balance within neural circuits and is required for contextual memory in the hyppocampus (By similarity). Plays a key role in the structural and functional plasticity of neurons (By similarity). Acts as an early-response transcription factor in both excitatory and inhibitory neurons, where it induces distinct but overlapping sets of late-response genes in these two types of neurons, allowing the synapses that form on inhibitory and excitatory neurons to be modified by neuronal activity in a manner specific to their function within a circuit, thereby facilitating appropriate circuit responses to sensory experience. [...]
9983	Achaete-scute homolog 2	AS-C proteins are involved in the determination of the neuronal precursors in the peripheral nervous system and the central nervous system.
14018	Fer3-like protein	Transcription factor that binds to the E-box and functions as inhibitor of transcription. DNA binding requires dimerization with an E protein. Inhibits transcription activation by ASCL1/MASH1 by sequestering E proteins.

Supplementary Table 10: **Name and function of bilaterian-specific transcription factors with homeobox and bHLH domain.** Left and middle column indicate orthogroup ID and full name of the corresponding human ortholog according to UniProt. Right column shows description of the respective factor as provided by UniProt. [...] indicates that part of the description has been omitted.

OG	Name	Function	Expression
OG_13067	Transmembrane protein 169	multi-pass membrane protein, function unknown	D: CNS, ventral nerve cord, ventral midline, brain; URL: http://insitu.fruitfly.org/cgi-bin/ex/report.pl?ftype=1&ftext=FBgn0037849
			M: CNS and different brain regions; URL: https://www.ebi.ac.uk/gxa/genes/ENSMUSG00000026188?bs=%7B%22mus%20musculus%22%3A%5B%22ORGANISM_PART%22%5D%7D&ds=%7B%22kingdom%22%3A%5B%22animals%22%5D%7D#baseline
OG_26661	Transmembrane protein 74B	multi-pass membrane protein, function unknown	D: not present in Ecdysozoa
			M: low expression in different tissues; URL: https://www.ebi.ac.uk/gxa/genes/ENSMUSG00000044364?bs=%7B%22mus%20musculus%22%3A%5B%22ORGANISM_PART%22%5D%7D&ds=%7B%22kingdom%22%3A%5B%22animals%22%5D%7D#baseline
OG_28197	Transmembrane protein 160	multi-pass membrane protein, function unknown	D: not present in Ecdysozoa
			M: CNS and different brain regions; URL: https://www.ebi.ac.uk/gxa/genes/ENSMUSG00000019158?bs=%7B%22mus%20musculus%22%3A%5B%22ORGANISM_PART%22%5D%7D&ds=%7B%22kingdom%22%3A%5B%22animals%22%5D%7D#baseline

Supplementary Table 11: **Name and expression of bilaterian-specific transmembrane proteins with unknown function.** First and second column indicate orthogroup ID and full name of the corresponding human ortholog according to UniProt. Right column shows expression data as provided by the Berkeley Drosophila Genome Project for *D. melanogaster* (D) [Tomancak et al. 2002] and the EMBL Expression Atlas for *Mus musculus* (M) [Petryszak et al. 2016]. CNS: central nervous system. URLs link directly to the relevant expression data.

OG	Name	OG comp.	HMM hits	# Species	Ancestor	E-value
OG_13067	TM169/CG4596	27 D; 9 L; 23 E	1. OG_13067	59	Bilateria	$4.0e^{-126}$
			2. OG_761179	2	Eumetazoa	2.0
			3. OG_320755	3	Eumetazoa	2.4
			4. OG_522863	2	*Acropora*	5.3
OG_28197	TM160	22 D; 8 L; 0 E	1. OG_28197	30	Bilateria	$1.0e^{-103}$
			2. OG_453098	2	Sarcopterygii	$2.6e^{-17}$
			3. OG_64229	15	Opisthokonta	0.00056
			4. OG_17167	54	Fungi	0.32

Supplementary Table 12: HMM-HMM search results for two transmembrane proteins with unknown function. Orthogroup ID, corresponding gene name, and orthogroup composition is shown for two bilaterian-specific transmembrane proteins without described function. Columns 4–7 summarise results of HMM-HMM searches in our database. In column four and five, orthogroup ID and corresponding species number of the three best search hits for each query are shown. Column six and seven report last common ancestor and corresponding E-value of hit orthogroups. D = Deuterostomia; E = Ecdysozoa; L = Lophotrochozoa; n.d. = not determined.

OG	Name	OG comp.	HMM hits	# Species	Ancestor	E-value
OG_26631	CG13034	2 Ch; 1 My; 2 Cr; 23 He	1. OG_26631	28	Arthropoda	$4e^{-134}$
			2. OG_402434	2	Endopterygota	$2.7e^{-32}$
			3. OG_22344	30	Panarthropoda	$9.8e^{-08}$
			4. OG_62779	13	Arthropoda	0.34
OG_34551	CG10433	4 Ch; 1 My; 2 Cr; 19 He	1. OG_34551	26	Arthropoda	$9.6e^{-56}$
			2. OG_330852	3	Formicidae	$7.8e^{-10}$
			3. OG_490587	2	Obtectomera	$1.4e^{-07}$
			4. OG_291047	3	Neognathae	0.007
OG_35928	CG16926	2 Ch; 1 My; 0 Cr; 21 He	1. OG_35928	24	Arthropoda	$1.4e^{-52}$
			2. OG_800521	2	Trichoderma	2.6
			3. OG_807347	2	Rhizoctonia	3
			4. OG_430491	2	Gnathostomata	3.5

Supplementary Table 13: HMM-HMM search results for three uncharacterised arthropod-specific proteins. Orthogroup ID, corresponding *D. melanogaster* gene name, and orthogroup composition is shown for three arthropod-specific proteins without described function. Columns 4–7 summarise results of HMM-HMM searches in our database. In column four and five, orthogroup ID and species number within the four best search hits for each query are shown. Column six and seven report last common ancestor and corresponding E-value of orthogroup hits. Ch = Chelicerata; Cr = Crustacea; He = Hexapoda; My = Myriapoda. Note that each query HMM detects itself as best hit. All next similar HMM-HMM hits correspond either to orthogroups within Arthropoda (Endopterygota, Panarthropoda, Formicidae, Obtectomera) or to very small, phylogenetically distant orthogroups outside arthropods with low similarity (Neognathae, Trichoderma, Rhizoctonia, Gnathostomata), illustrating that these arthropod-specific proteins do not possess related domains outside their lineage. For corresponding multiple sequence alignments, see Figure 4–Figure Supplement 1.

OG	Name	OG comp.	HMM hits	Name	Ancestor	OG composition
OG_10143	Eomes	49 D; 4 L; 2 E	1. OG_10143	Eomes	Bilateria	49 D; 4 L; 2 E
			2. OG_8510	T	Holozoa	1 Ic; 1 Pl; 3 Po; 6 Cn; 6 Ct; 73 B
			3. OG_242	TBX2	Metazoa	1 Pl; 4 Po; 14 Cn; 8 Ct; 127 B
			4. OG_13145	MGAP	Gnathost.	40
OG_10785	EGF-CFC	52 D; 3 L; 0 E	1. OG_10785	EGF-CFC	Bilateria	52 D; 3 L; 0 E
			2. OG_250903	n.d.	Euteleost.	3
			3. OG_124091	n.d.	Gnathost.	4
			4. OG_408474	n.d.	Echinoida	2
OG_11821	Lefty	52 D; 3 L; 0 E	1. OG_11821	Lefty	Bilateria	52 D; 3 L; 0 E
			2. OG_195649	n.d.	Gnathost.	3
			3. OG_122960	n.d.	Bilateria	5 D; 1 L
			4. OG_580	GDF3	Metazoa	1 Pl; 2 Po; 15 Cn; 8 Ct; 130 B
OG_12210	Nodal	44 D; 6 L; 0 E	1. OG_12210	Nodal	Bilateria	44 D; 6 L; 0 E
			2. OG_580	GDF3	Metazoa	1 Pl; 2 Po; 15 Cn; 8 Ct; 130 B
			3. OG_9136	GDF6	Metazoa	2 Po; 10 Cn; 53 B
			4. OG_9694	GDF2	Bilateria	45 D; 5 L; 6 E
OG_36001	FoxH1	23 D; 2 L; 0 E	1. OG_36001	FOXH1	Bilateria	23 D; 2 L; 0 E
			2. OG_63374	FOXH1-2	Bilateria	13 D; 1 E
			3. OG_302079	n.d.	Ct	2
			4. OG_3972	FOXD4	Metazoa	1 Pl; 4 Po; 8 Cn; 7 Ct; 109 B
OG_63374	FoxH1-2	13 D; 0 L; 1 E	1. OG_63374	FoxH1-2	Bilateria	13 D; 0 L; 1 E
			2. OG_36001	FoxH1	Bilateria	23 D; 2 L; 0 E
			3. OG_11471	FoxI1	Metazoa	1 Po; 48 D; 0 E; 1 L
			4. OG_8725	FoxL2	Metazoa	5 Po; 3 Ct; 12 Cn; 75 B

Supplementary Table 14: HMM-HMM search results for Nodal pathway members. Orthogroup ID, corresponding gene name, and orthogroup composition is shown for the five bilaterian-specific Nodal pathway genes. Columns 4–7 summarise results of HMM-HMM searches in our database. In column four and five, orthogroup ID and corresponding gene name of the three best search hits for each Nodal pathway member are shown. Column six and seven report last common ancestor and composition of hit orthogroups. Abbreviations: B = Bilateria; Cn = Cnidaria; Ct = Ctenophora; D = Deuterostomia; E = Ecdysozoa; Ic = Ichthyosporea; L = Lophotrochozoa; Pl = Placozoa; Po = Porifera. Euteleost. = Euteleostomi; Gnathost. = Gnathostomata; n.d. = not determined; T = Brachyury; TBX = T-box protein; MGAP = MAX gene-associated protein; GDF= Growth/differentiation factor; FOX= Forkhead box protein. Holozoa is common ancestor of Metazoa + Choanoflagellida + Ichthyosporea. OG_63374 is reciprocal best hit of OG_36001 (FoxH1) in HMM-HMM searches.

<table>
<tr><th>OG</th><th>Name</th><th>Outlier SeqID</th><th>4 Best hits at NCBI in Bilateria</th><th>E-value</th></tr>
<tr><td>OG_5226</td><td>Odd-skipped-related (OSR)</td><td>45264|trs_EZ022029.1_8_0</td><td>1. XP_003512540.3 (zinc finger protein 431 [Cricetulus griseus])</td><td>$2.0e^{-14}$</td></tr>
<tr><td></td><td></td><td></td><td>2. XP_027262642.1 (zinc finger protein 431 [Cricetulus griseus])</td><td>$2.0e^{-14}$</td></tr>
<tr><td></td><td></td><td></td><td>3. XP_006898891.1 (zinc finger protein 699-like [Elephantulus edwardii])</td><td>$4.0e^{-14}$</td></tr>
<tr><td></td><td></td><td></td><td>4. XP_024655672.1 (zinc finger protein 660-like [Maylandia zebra]</td><td>$5.0e^{-14}$</td></tr>
<tr><td></td><td></td><td>747676|gi_328860070</td><td>1. 1LLM_C (chimera of Zif23-GCN4 [Mus musculus])</td><td>$2.0e^{-08}$</td></tr>
<tr><td></td><td></td><td></td><td>2. KOF92858.1 (OCBIM_22005869mg [Octopus bimaculoides])</td><td>$6.0e^{-07}$</td></tr>
<tr><td></td><td></td><td></td><td>3. XP_017758395.1 (zinc finger E-box-binding [Eufriesea mexicana])</td><td>$7.0e^{-07}$</td></tr>
<tr><td></td><td></td><td></td><td>4. XP_017758394.1 (tramtrack [Eufriesea mexicana])</td><td>$7.0e^{-07}$</td></tr>
<tr><td></td><td></td><td>1126212|gi_407917167</td><td>1. XP_008474334.2 (zinc finger protein 570-like [Diaphorina citri])</td><td>$1.0e^{-10}$</td></tr>
<tr><td></td><td></td><td></td><td>2. XP_030058979.1 (E3 SUMO-protein ligase EGR2 [Microcaecilia unicolor])</td><td>$5.0e^{-10}$</td></tr>
<tr><td></td><td></td><td></td><td>3. XP_029908092.1 (Sp7 isoform X1 [Myripristis murdjan])</td><td>$6.0e^{-10}$</td></tr>
<tr><td></td><td></td><td></td><td>4. XP_029908093.1 (Sp7 isoform X2 [Myripristis murdjan])</td><td>$7.0e^{-10}$</td></tr>
<tr><td>OG_5717</td><td>Slit</td><td>10228|gi_190582737</td><td>1. XP_028966320.1 (protein slit [Galendromus occidentalis])</td><td>0.0</td></tr>
<tr><td></td><td></td><td></td><td>2. ELU02319.1 (CAPTEDRAFT_179696 [Capitella teleta])</td><td>0.0</td></tr>
<tr><td></td><td></td><td></td><td>3. XP_013386132.1 (slit homolog 2 [Lingula anatina])</td><td>0.0</td></tr>
<tr><td></td><td></td><td></td><td>4. AAI70476.1 (Slit2-a protein [Xenopus laevis])</td><td>0.0</td></tr>
<tr><td>OG_10143</td><td>Eomes</td><td>A0A077SN37_9METZ</td><td>1. XP_012940912.1 (TBX6L-like, partial [Aplysia californica])</td><td>$2.0e^{-57}$</td></tr>
<tr><td></td><td></td><td></td><td>2. XP_013379423.1 (TBX3 isoform X3 [Lingula anatina])</td><td>$7.0e^{-57}$</td></tr>
<tr><td></td><td></td><td></td><td>3. XP_013379419.1 (TBX3 isoform X1 [Lingula anatina])</td><td>$3.0e^{-56}$</td></tr>
<tr><td></td><td></td><td></td><td>4. ELT90467.1 (CAPTEDRAFT_110717 [Capitella teleta])</td><td>$6.0e^{-56}$</td></tr>
<tr><td></td><td></td><td>Eomes_Leucosolenia from Sebé-Pedrós et al. [2013]</td><td>1. XP_012403491.1 (TBX4 [Sarcophilus harrisii])</td><td>$3.0e^{-58}$</td></tr>
<tr><td></td><td></td><td></td><td>2. TWW77798.1 (TBX4 [Takifugu flavidus])</td><td>$3.0e^{-57}$</td></tr>
<tr><td></td><td></td><td></td><td>3. XP_020847689.1 (TBX4 isoform X1 [Phascolarctos cinereus])</td><td>$3.0e^{-57}$</td></tr>
<tr><td></td><td></td><td></td><td>4. XP_027554579.1 (TBX4 [Neopelma chrysocephalum])</td><td>$4.0e^{-57}$</td></tr>
</table>

Supplementary Table 15: RBH Blast search for potential outliers. First and second column: Orthogroup identifier in BigWenDB and corresponding gene name. Third column: Sequence ID of potential outliers used as query for reciprocal best hit Blast search. Fourth and fifth column show the four best hits when performing Blast searches in the non-redundant protein database at NCBI within the taxon Bilateria, and corresponding E-values.

OG	Name	OG comp.	HMM hits	Name	Ancestor	OG composition
OG_12210	Nodal	44 D; 6 L; 0 E	1. OG_12210	Nodal	Bilateria	44 D; 6 L; 0 E
			2. OG_580	GDF3	Metazoa	1 Pl; 2 Po; 15 Cn; 8 Ct; 130 B
			3. OG_9136	GDF6	Metazoa	2 Po; 10 Cn; 53 B
			4. OG_9694	GDF2	Bilateria	45 D; 5 L; 6 E
OG_9136	GDF6	2 Po; 10 Cn; 53 B	1. OG_9136	GDF6	Metazoa	2 Po; 10 Cn; 53 B
			2. OG_580	GDF3	Metazoa	1 Pl; 2 Po; 15 Cn; 8 Ct; 130 B
			3. OG_9694	GDF2	Bilateria	45 D; 5 L; 6 E
			4. OG_293907	n.d.	Ambulacraria	3
OG_9136_Cn	GDF6_Cn	10 Cn	1. OG_9136	GDF6	Metazoa	2 Po; 10 Cn; 53 B
			2. OG_580	GDF3	Metazoa	1 Pl; 2 Po; 15 Cn; 8 Ct; 130 B
			3. OG_562159	n.d.	Hexacorallia	2
			4. OG_9694	GDF2	Bilateria	45 D; 5 L; 6 E

Supplementary Table 16: HMM-HMM search results for a putative Hydra Nodal-related gene. Orthogroup ID, corresponding gene name, and orthogroup composition is shown for Nodal and a *Hydra magnipapillata* Nodal-related gene as published in [Watanabe et al. 2014]. Columns 4–7 summarise results of HMM-HMM searches in our database. In column four and five, orthogroup ID and corresponding gene name of the four best search hits for each query are shown. Column six and seven report last common ancestor and composition of hit orthogroups. Abbreviations: B = Bilateria; Cn = Cnidaria; Ct = Ctenophora; D = Deuterostomia; E = Ecdysozoa; L = Lophotrochozoa; Pl = Placozoa; Po = Porifera; n.d. = not determined; GDF= Growth/differentiation factor. Note that each query HMM detects itself as best hit. OG_12210 (Nodal), OG_9136 (containing *Hydra* Nodal-related), and OG_9136_Cn, containing only cnidarian Nodal-related genes, are not engaged in a reciprocal best hit relationship in HMM-HMM searches, arguing against a common evolutionary origin.

OG	Name	OG comp.	HMM hits	OG comp.	Ancestor	E-value
OG_278	5HT1A_HUMAN	D 55; E 44; L 14	1. OG_278	D 55; E 44; L 14	Bilateria	$9.0e^{-115}$
	5-hydroxy-		2. OG_38825	D 14; E 3; L 1	Bilateria	$4.1e^{-74}$
	tryptamine		3. OG_20041	D 1; E 25; L 2	Bilateria	$6.7e^{-72}$
	receptor 1A		4. OG_87181	D 6	Deuterost.	$2.0e^{-70}$
OG_1451	SSR5_HUMAN	D 59; E 44; L 10	1. OG_1451	D 59; E 44; L 10	Bilateria	$2.0e^{-166}$
	Somatostatin		2. OG_197068	2	Gnathost.	$3.5e^{-84}$
	receptor		3. OG_17701	Ct 1; D 34	Eumeta.	$2.3e^{-81}$
	type 5		4. OG_22358	D 28	Deuterost.	$6.3e^{-77}$
OG_6712	DRD2_HUMAN	D 47; E 33; L 7	1. OG_6712	D 47; E 33; L 7	Bilateria	$1.0e^{-252}$
	D(2) dopamine		2. OG_87181	D 6	Deuterost.	$1.5e^{-88}$
	receptor		3. OG_91943	8	Nematoda	$9.0e^{-88}$
			4. OG_20041	D 1; E 25; L 2	Bilateria	$1.7e^{-87}$
OG_6895	SCTR_HUMAN	D 53; E 12; L 9	1. OG_6895	D 53; E 12; L 9	Bilateria	$2.0e^{-246}$
	Secretin re-		2. OG_7679	D 52; E 0; L 2	Bilateria	$2.0e^{-124}$
	ceptor		3. OG_8480	D 53; E 1; L 0	Bilateria	$7.0e^{-108}$
			4. OG_23947	36	Gnathost.	$1.0e^{-102}$
OG_7773	CRFR2_HUMAN	D 39; E 38; L 11	1. OG_7773	D 39; E 38; L 11	Bilateria	$1.0e^{-176}$
	Corticotropin-		2. OG_6895	D 53; E 12; L 9	Bilateria	$5.8e^{-87}$
	releasing factor		3. OG_13184	Cn 1; D 6; E 40; L 7	Eumeta.	$1.0e^{-83}$
	receptor 2		4. OG_80843	D 1; E 9; L 0	Bilateria	$4.8e^{-83}$
OG_8006	CALCR_HUMAN	D 44; E 31; L 9	1. OG_8006	D 44; E 31; L 9	Bilateria	$4.0e^{-152}$
	Calcitonin re-		2. OG_110397	8	Gnathost.	$1.0e^{-93}$
	ceptor		3. OG_20497	30	Protost.	$7.4e^{-83}$
			4. OG_7773	D 39; E 38; L 11	Bilateria	$1.2e^{-74}$
OG_16379	NMUR2_HUMAN	D 9; E 15; L 12	1. OG_16379	D 9; E 15; L 12	Bilateria	$6.5e^{-54}$
	Neuromedin-U		2. OG_3925	Cn 2; D 51; E 41; L 8	Eumeta.	$5.5e^{-35}$
	receptor 2		3. OG_135144	D 2; E 4; L 0	Bilateria	$1.4e^{-30}$
			4. OG_155343	D 4; E 1; L 0	Bilateria	$1.0e^{-25}$
OG_34259	ADA2B_HUMAN	D 7; E 10; L 6	1. OG_34259	D 7; E 10; L 6	Bilateria	$1.1e^{-69}$
	Alpha-2B		2. OG_23231	Cn 2; D 3; E 18; L 6	Eumeta.	$5.9e^{-46}$
	adrenergic		3. OG_38825	D 14; E 3; L 1	Bilateria	$8.1e^{-43}$
	receptor		4. OG_87181	D 6	Deuterost.	$2.1e^{-41}$

Supplementary Table 17: HMM-HMM search results for bilaterian-specific G protein-coupled receptors. Orthogroup ID, corresponding human gene name (UniProt ID), and orthogroup composition is shown for eight bilaterian-specific G protein-coupled receptor proteins. Columns 4–7 summarise results of HMM-HMM searches. Columns four and five show orthogroup ID and composition of the four best search hits for each query HMM. Columns six and seven report their last common ancestor and corresponding E-value. Cn = Cnidaria; Ct = Ctenophora; D/Deuterost. = Deuterostomia; E = Ecdysozoa; Eumeta. = Eumetazoa; Gnathost. = Gnathostomata; L = Lophotrochozoa; Protost. = Protostomia. Note that each query HMM detects itself as best hit. Most next similar hits correspond to orthogroups within Bilateria (Deuterostomia, Gnathostomata, Nematoda, Protostomia). Four HMM-HMM hits belong to more ancient orthogroups with the potential to shift the inferred bilaterian ancestor (highlighted in red), but in all these cases hit orthogroup composition does not support a (eu)metazoan ancestor on a broad phylogenetic basis as only one or two non-bilaterian species are present. In addition, the reciprocal best hit criterion is not fulfilled in all but one case (OG_23231), arguing that most of the eight GPCRs originated in the ancestor of bilaterians.

OG	OG comp.	HMM hits	OG comp.	Ancestor	E-value
OG_17701	Ct 1; D 34	1. OG_17701	Ct 1; D 34	Metazoa	$8.0e^{-183}$
		2. OG_161673	4	Sarcopterygii	$8.0e^{-118}$
		3. OG_1451	D 59; E 44; L 10	Bilateria	$2.4e^{-84}$
		4. OG_95038	9	Gnathostomata	$3.2e^{-81}$
OG_13184	Cn 1; D 6; E 40; L 7	1. OG_13184	Cn 1; D 6; E 40; L 7	Eumetazoa	$5.0e^{-264}$
		2. OG_7773	D 39; E 38; L 11	Bilateria	$2.6e^{-84}$
		3. OG_35449	Cn 6; D 3; E 10; L 3	Eumetazoa	$1.3e^{-82}$
		4. OG_6895	D 53; E 12; L 9	Bilateria	$6.5e^{-78}$
OG_3925	Cn 2; D 51; E 41; L 8	1. OG_3925	Cn 2; D 51; E 41; L 8	Eumetazoa	$1.0e^{-139}$
		2. OG_14939	Cn 1; D 45; E 1	Eumetazoa	$3.7e^{-76}$
		3. OG_187053	D 2; L 2	Bilateria	$3.1e^{-61}$
		4. OG_2778	Pl 1; Po 1; B 105	Metazoa	$2.5e^{-60}$
OG_23231	Cn 2; D 3; E 18; L 6	1. OG_23231	Cn 2; D 3; E 18; L 6	Eumetazoa	$4.6e^{-98}$
		2. OG_34259	D 7; E 10; L 6	Bilateria	$1.2e^{-45}$
		3. OG_38825	D 14; E 3; L 1	Bilateria	$9.6e^{-40}$
		4. OG_1412	Pl 1; Cn 2; B 111	Metazoa	$1.1e^{-38}$

Supplementary Table 18: Reverse HMM-HMM search results for GPCR hits with metazoan ancestor. Orthogroup ID and composition is shown for four orthogroups of Supplementary Table 17 with a best HMM-HMM hit relationship to bilaterian-specific GPCRs and origin prior to the bilaterian ancestor. Columns 3–6 summarise results of HMM-HMM searches in our database. In column three and four, orthogroup ID and composition of the four best search hits for each query HMM are shown. Columns five and six report their last common ancestor and corresponding E-value. B = Bilateria; Cn = Cnidaria; Ct = Ctenophora; D = Deuterostomia; E = Ecdysozoa; L = Lophotrochozoa; Pl = Placozoa; Po = Porifera. Note that each query HMM detects itself as best hit. The HMM-HMM analysis indicates that no reciprocal best-hit orthogroups for the bilaterian-specific GPCRs of Supplementary Table 17 exist in non-bilaterian metazoans, supporting the bilaterian origin of these GPCRs.

OG	Name	OG comp.	HMM hits	OG comp.	Ancestor	E-value
OG_3707	Netrin	Pl 1; Po 1; Cn 10 D 55; E 41; L 13	1. OG_3707	Pl 1; Po 1; Cn 10 D 55; E 41; L 13	Metazoa	$2.0e^{-119}$
			2. OG_19436	Po 1; Cn 3; D 11 E 11; L 10	Metazoa	$2.2e^{-84}$
			3. OG_4825	Pl 1; Po 2; Ct 4 Cn 8; D 60; E 40 L 10	Metazoa	$1.4e^{-76}$
			4. OG_9036	Cn 8; D 44; E 1 L 4	Eumetazoa	$5.0e^{-41}$
OG_5220	DCC, Deleted in Colorectal Cancer	Pl 1; Po 1; Cn 12 D 44; E 44; L 11	1. OG_5220	Pl 1; Po 1; Cn 12 D 44; E 44; L 11	Metazoa	$0.0e^{0}$
			2. OG_5970	Po 1; Cn 6; D 54 E 15; L 7	Metazoa	$3.0e^{-128}$
			3. OG_4128	Pl 1; Cn 1; D 52 E 39; L 8	Metazoa	$1.6e^{-88}$
			4. OG_5220	Pl 1; Po 1; Cn 12 D 44; E 44; L 11	Metazoa	$4.6e^{-83}$
OG_5717	Slit	Pl 1; D 46; E 41 L 12	1. OG_5717	Pl 1; D 46; E 41 L 12	Metazoa	$7.0e^{-249}$
			2. OG_59323	Po 1; D 4; E 6 L 5	Metazoa	$1.1e^{-68}$
			3. OG_5717	Pl 1; D 46; E 41 L 12	Metazoa	$5.9e^{-61}$
			4. OG_6995	Cn 1; D 35; E 48 L 13	Eumetazoa	$1.6e^{-53}$
OG_4128	Robo, Round-About	Pl 1; Cn 1; D 52 E 39; L 8	1. OG_4128	Pl 1; Cn 1; D 52 E 39; L 8	Metazoa	$0.0e^{0}$
			2. OG_153953	Cn 1; L 3	Eumetazoa	$4.0e^{-121}$
			3. OG_11356	Cn 1; D 49	Eumetazoa	$6.0e^{-119}$
			4. OG_135605	Cn 1; E 5	Eumetazoa	$6.0e^{-111}$
			5. OG_51853	Cn 9	Cnidaria	$1.0e^{-95}$
OG_51853	Cnidarian Robo	Cnidaria 9	1. OG_51853	Cn 9	Cnidaria	$0.0e^{0}$
			2. OG_4128	Pl 1; Cn 1; D 52 E 39; L 8	Metazoa	$1.1e^{-98}$
			3. OG_232708	3	Hydrozoa	$1.1e^{-67}$
			4. OG_5970	Po 1; Cn 6; D 54 E 15; L 7	Metazoa	$2.1e^{-60}$

Supplementary Table 19: HMM-HMM search results for two major axon guidance pathways. Orthogroup ID, corresponding gene name, and orthogroup composition is shown for the Netrin-DCC and Slit-Robo axon guidance molecules. Columns 4–7 summarise results of HMM-HMM searches in our database. In column four and five, orthogroup ID and composition of the four best search hits for each query HMM are shown. Columns six and seven report their last common ancestor and corresponding E-value. Cn = Cnidaria; Ct = Ctenophora; D = Deuterostomia; E = Ecdysozoa; L = Lophotrochozoa; Pl = Placozoa; Po = Porifera. Note that each query HMM detects itself as best hit. OG_51853 is the reciprocal best hit in cnidarians (green highlight) of Robo orthogroup OG_4128, suggesting a (eu)metazoan origin of this receptor. Similarly, the composition of the Netrin and DCC orthogroups and of their HMM search hits suggests a pre-bilaterian origin of these factors.

OG	Name	OG comp.	Description
OG_2290	ENAH_HUMAN	Pl 1; Po 3; Ct 8; Cn 12; B 122	Ena/VASP proteins are actin-associated proteins involved in a range of processes dependent on cytoskeleton remodeling and cell polarity such as axon guidance and lamellipodial and filopodial dynamics in migrating cells. ENAH induces the formation of F-actin rich outgrowths in fibroblasts. Acts synergistically with BAIAP2-alpha and downstream of NTN1 to promote filipodia formation (By similarity).
	ENA_DROME		Functions, together with Abl, trio, and fra, in a complex signalling network that regulates axon guidance at the CNS midline. Required in part for Robo-mediated repulsive axon guidance. May be involved in lamellipodial dynamics.
OG_1379	SOS1_HUMAN	F 77; Pl 1; Po 5; Ct 9; Cn 14; B 101	Promotes the exchange of Ras-bound GDP by GTP (PubMed:8493579). Probably by promoting Ras activation, regulates phosphorylation of MAP kinase MAPK3 in response to EGF (PubMed:17339331). Catalytic component of a trimeric complex that participates in transduction of signals from Ras to Rac by promoting the Rac-specific guanine nucleotide exchange factor (GEF) activity (By similarity).
	SOS_DROME		Promotes the exchange of Ras-bound GDP by GTP. Functions in signalling pathways initiated by the sevenless and epidermal growth factor receptor tyrosine kinases; implies a role for the ras pathway in neuronal development.

Supplementary Table 20: Ancient origin of Robo downstream signalling components. Orthogroup ID, corresponding gene name, orthogroup composition, and available information is shown for two known members of the Robo downstream signalling cascade, the Ena/VASP protein "Enabled" and the guanyl-nucleotide exchange factor "Son of sevenless". Description is taken from UniProt. B = Bilateria; Cn = Cnidaria; Ct = Ctenophora; F = Fungi; Pl = Placozoa; Po = Porifera.

OG	Name	OG comp.	HMM hits	OG comp.	Ancestor	E-value
OG_5717	Slit	Pl 1; B 103	1. OG_5717	Pl 1; B 103	Metazoa	$7.0e^{-249}$
			2. OG_59323	Po 1; B 16	Metazoa	$1.1e^{-68}$
			3. OG_5717	Pl 1; B 103	Metazoa	$5.9e^{-61}$
			4. OG_6995	Cn 1; B 96	Eumetazoa	$1.6e^{-53}$
OG_59323		Po 1; B 16	1. OG_59323	Po 1; B 16	Metazoa	$3.0e^{-166}$
			2. OG_5717	Pl 1; B 103	Metazoa	$6.1e^{-71}$
			3. OG_48441	Cn 4; B 13	Eumetazoa	$1.6e^{-30}$
			4. OG_7809	Pl 1; Po 4; Cn 11; B 33	Metazoa	$7.7e^{-29}$
OG_6995		Cn 1; B 96	1. OG_6995	Cn 1; B 96	Eumetazoa	$2.0e^{-160}$
			2. OG_59370	D 3; E 0; L 1	Bilateria	$8.8e^{-89}$
			3. OG_10044	D 37; E 0; L 4	Bilateria	$1.4e^{-87}$
			4. OG_8851	Cn 1; B 61	Eumetazoa	$2.2e^{-84}$

Supplementary Table 21: HMM-HMM search results for the bilaterian-specific Slit orthogroup. Orthogroup ID, corresponding human gene name, and orthogroup composition is shown for the Slit orthogroup and two of its HMM search hits. Columns 4–7 summarise results of HMM-HMM searches in our database. In column four and five, orthogroup ID and composition of the four best search hits for each query HMM are shown. Columns six and seven report their last common ancestor and corresponding E-value. Cn = Cnidaria; D = Deuterostomia; E = Ecdysozoa; L = Lophotrochozoa; Pl = Placozoa; Po = Porifera. OG_59323 contains genomic and transcriptomic ORFs of 16 bilaterian species and a sponge and is the reciprocal best hit of Slit OG_5717. Its single outlier, a sequence from the sponge *Crella elegans*, belongs to the EGF superfamily and is more similar to protocadherins and Notch than to Slit. The other orthogroup members are Slit protein fragments that were not included into Slit OG_5717. These HMM-HMM comparisons support the bilaterian ancestry of Slit.

OG	Name	OG comp.	HMM hits	OG comp.	Ancestor	E-value
OG_14798	NGF, Nerve Growth Factor	D 37; E 3; L 2	1. OG_14798	D 37; E 3; L 2	Bilat	$2.0e^{-138}$
			2. OG_21801	D 26; E 2; L 4	Bilat	$3.0e^{-54}$
			3. OG_113491	D 2; E 1; L 4	Bilat	$2.4e^{-18}$
			4. OG_481165	2	Deuterost	$4.7e^{-09}$
OG_21801	BDNF, Brain-derived Neurotrophic Factor	D 26; E 2; L 4	1. OG_21801	D 26; E 2; L 4	Bilat	$8.0e^{-240}$
			2. OG_14798	D 37; E 3; L 2	Bilat	$6.7e^{-54}$
			3. OG_113491	D 2; E 1; L 4	Bilat	$3.7e^{-16}$
			4. OG_5071	Pl 1; Po 6; Cn 12 Ct 9; B 103	Opisthok	$2.8e^{-10}$
OG_14798	NT3, Neuro-trophin 3	D 37; E 3; L 2	1. OG_14798	D 37; E 3; L 2	Bilat	$2.0e^{-138}$
			2. OG_21801	D 26; E 2; L 4	Bilat	$3.0e^{-54}$
			3. OG_113491	D 2; E 1; L 4	Bilat	$2.4e^{-18}$
			4. OG_481165	2	Deuterost	$4.7e^{-09}$
OG_21801	NT4, Neuro-trophin 4	D 26; E 2; L 4	1. OG_21801	D 26; E 2; L 4	Bilat	$8.0e^{-240}$
			2. OG_14798	D 37; E 3; L 2	Bilat	$6.7e^{-54}$
			3. OG_113491	D 2; E 1; L 4	Bilat	$3.7e^{-16}$
			4. OG_5071	Pl 1; Po 6; Cn 12 Ct 9; B 103	Opisthok	$2.8e^{-10}$
OG_20167	CNTF, Ciliary Neurotrophic Factor	Euteleost 34	1. OG_20167	34	Euteleost	$9.0e^{-248}$
			2. OG_124946	5	Clupeoceph	$1.0e^{-126}$
			3. OG_578800	2	Pseudocreni	$1.3e^{-95}$
			4. OG_455713	2	Sarcopter	$5.2e^{-93}$
OG_20569	IGF-1, Insulin-like Growth Factor 1	D 37; E 4; L 0	1. OG_20569	D 37; E 4; L 0	Bilat	$2.0e^{-122}$
			2. OG_36002	25	Gnathost	$3.6e^{-28}$
			3. OG_197349	4	Gnathost	$1.2e^{-21}$
			4. OG_438317	2	Gnathost	$9.3e^{-19}$
OG_36002	IGF-2, Insulin-like Growth Factor 2	Gnathost 25	1. OG_36002	25	Gnathost	$2.0e^{-122}$
			2. OG_276483	3	Gnathost	$2.2e^{-36}$
			3. OG_20569	D 37; E 4; L 0	Bilat	$1.4e^{-28}$
			4. OG_427655	2	Gnathost	$4.1e^{-26}$

Supplementary Table 22: HMM-HMM search results for neurotrophic growth factors, part 1. Orthogroup ID, corresponding human gene name, and orthogroup composition is shown for the known vertebrate neurotrophic growth factors. Columns 4–7 summarise results of HMM-HMM searches in our database. In column four and five, orthogroup ID and composition of the four best search hits for each query HMM are shown. Columns six and seven report their last common ancestor and corresponding E-value. B/Bilat = Bilateria; Clupeoceph = Clupeocephala; Cn = Cnidaria; Ct = Ctenophora; D/Deuterost. = Deuterostomia; E = Ecdysozoa; Euteleost = Euteleostomi; Gnathost. = Gnathostomata; L = Lophotrochozoa; Opisthok = Opisthokonta; Pl = Placozoa; Po = Porifera; Pseudocreni = Pseudocrenilabrinae; Sarcopter = Sarcopterygii. Note that each query HMM detects itself as best hit. In most cases, the next similar HMM hits correspond to orthogroups within Bilateria (e. g. Deuterostomia, Gnathostomata, Sarcopterygii). Exceptions are OG_5071, which constitutes an evolutionary old component of the R2TP complex that is involved in snoRNP biogenesis and RNA polymerase II assembly; OG_5812, that belongs to the PDGF/VEGF family and originated in the eumetazoan ancestor; and OG_119370, that contains TGF-β family members of cnidarians. These HMM-HMM comparisons could not reveal factors related to neurotrophins in non-bilaterian metazoans, arguing that neurotrophic growth factors are a bilaterian innovation and diversified further during vertebrate evolution (see Figure 7, and Supplementary Table 23 for part 2 of the table).

OG	Name	OG comp.	HMM hits	OG comp.	Ancestor	E-value
OG_36013	LIF,	Sarcopter 25	1. OG_36013	25	Sarcopter	$8.9e^{-80}$
	Leukemia		2. OG_140291	6	Clupeoceph	0.00019
	Inhibitory		3. OG_33370	25	Euteleost	0.00044
	Factor		4. OG_34716	26	Eutheria	0.019
OG_28284	SCF,	Gnathost 24	1. OG_28284	24	Gnathost	$6.0e^{-156}$
	Stem Cell		2. OG_265601	2	Gnathost	$2.5e^{-20}$
	Factor		3. OG_95041	7	Neopterygii	$7.5e^{-17}$
			4. OG_138728	2	Gnathost	0.54
OG_10688	PDGF*,	D 46; E 1; L 0	1. OG_10688	D 46; E 1; L 0	Bilat	$2.0e^{-111}$
	Platelet-		2. OG_263502	3	Gnathost	$2.0e^{-14}$
	Derived Growth		3. OG_5812	Cn 17; Ct 0; B 62	Eumetazoa	$1.2e^{-12}$
	Factor		4. OG_263501	3	Gnathost	$1.7e^{-12}$
OG_32190	GDNF, Glial	Gnathost 22	1. OG_32190	22	Gnathost	$1.0e^{-122}$
	Cell Line-		2. OG_10692	47	Gnathost	$1.1e^{-33}$
	Derived Neuro-		3. OG_219414	3	Laurasiatheria	$3.8e^{-08}$
	trophic Factor		4. OG_119370	Cn 5; D 1; L 1	Eumetazoa	$9.0e^{-07}$
OG_10692	NTN,	Gnathost 47	1. OG_10692	47	Gnathost	$1.3e^{-79}$
	Neurturin		2. OG_32190	22	Gnathost	$2.4e^{-32}$
			3. OG_219414	3	Laurasiatheria	$2.5e^{-12}$
			4. OG_119370	Cn 5; D 1; L 1	Eumetazoa	$1.0e^{-05}$
OG_10692	PSP,	Gnathost 47	1. OG_10692	47	Gnathost	$1.3e^{-79}$
	Persephin		2. OG_32190	22	Gnathost	$2.4e^{-32}$
			3. OG_219414	3	Laurasiatheria	$2.5e^{-12}$
			4. OG_119370	Cn 5; D 1; L 1	Eumetazoa	$1.0e^{-05}$
OG_10867	NDNF,	D 31; E 22; L 9	1. OG_10867	D 31; E 22; L 9	Bilat	$5.0e^{-176}$
	Neuron-Derived		2. OG_93467	10	Diptera	$4.2e^{-28}$
	Neurotrophic		3. OG_210202	D 3; E 0; L 1	Bilat	$2.4e^{-27}$
	Factor		4. OG_70191	13	Endopterygota	$3.5e^{-24}$

Supplementary Table 23: HMM-HMM search results for neurotrophic growth factors, part 2. Caption see Supplementary Table 22. * The ecdysozoan sequence in this orthogroup is from *Priapulus caudatus*, very short, and unlike PDGF. A more correct ancestor for this group is therefore "Gnathostomata" (as summarized in Figure 7).

OG	Name	HMM hits	OG comp.	Ancestor	E-value
OG_67	Rtks and Rors	1. OG_67	Pl 1; Po 5; Ct 9; Cn 17; B 128	Opisthokonta	$2.0e^{-193}$
	n.d.	2. OG_13941	Po 3; Ct 1; B 5	Opisthokonta	$1.9e^{-87}$
	ROS fusion protein	3. OG_7862	Po 4; B 96	Opisthokonta	$4.0e^{-85}$
	Insulin receptor	4. OG_3768	Pl 1; Po 1; Cn 10; B 116	Opisthokonta	$8.9e^{-79}$
OG_8965-1.4	Rtks	1. OG_8965-1.4	D 45; E 6; L 8	Bilateria	n.d.
	Rtks and Rors	2. OG_67	Pl 1; Po 5; Ct 9; Cn 17; B 128	Opisthokonta	$6.6e^{-86}$
	n.d.	3. OG_53135	Po 1; Ct 1; B 14	Metazoa	$3.9e^{-84}$
	n.d.	4. OG_13941	Po 3; Ct 1; B 5	Opisthokonta	$1.6e^{-69}$
OG_6493-1.4	Rors	1. OG_6493-1.4	Po 4; Cn 12; B 96	Opis/Metazoa	n.d.
	Rtks and Rors	2. OG_67	Pl 1; Po 5; Ct 9; Cn 17; B 128	Opisthokonta	$8.0e^{-91}$
	n.d.	3. OG_13941	Po 3; Ct 1; B 5	Opisthokonta	$9.7e^{-78}$
	ROS fusion protein	4. OG_7862	Po 4; B 96	Opisthokonta	$2.3e^{-68}$

Supplementary Table 24: HMM-HMM search results for neurotrophin receptors of the Rtk family. Orthogroup ID, corresponding human gene name, and HMM-HMM search results (columns 3–6) are shown for neurotrophin receptors of the Rtk family, derived from two different clusterings with inflation parameter 1.3 (first block of rows) and 1.4 (second and third block of rows). In column three and four, orthogroup ID and composition of the four best search hits for each query HMM are shown; their corresponding gene name, if available, is indicated in column two. Columns five and six report their last common ancestor and corresponding E-value. B = Bilateria; Cn = Cnidaria; Ct = Ctenophora; D = Deuterostomia; E = Ecdysozoa; L = Lophotrochozoa; Opis = Opisthokonta; Pl = Placozoa; Po = Porifera. n.d. = not determined. While Rtks and Rors are erroneously combined in a large orthogroup with ancient origin (OG_67) under inflation parameter 1.3, this orthogroup is split in two parts with inflation parameter 1.4: a bilaterian-specific orthogroup containing Rtks (OG_8965-1.4) and a second orthogroup with more ancient origin containing Rors (OG_6493-1.4). Note that each query HMM detects itself as best hit.

#OGs	Taxon	Phylum
132,945	Ctenophora	Ctenophora
100,900	Eumetazoa	—
80,848	Gnathostomata	Vertebrata
76,441	Acropora	Cnidaria
48,993	Metazoa	—
47,457	Anthozoa	Cnidaria
**42,946**	**Bilateria**	—
32,196	Echinoida	Echinodermata
30,941	Sarcopterygii	Vertebrata
14,446	Opisthokonta	—
12,807	Hexacorallia	Cnidaria
12,154	Protostomia	—
10,387	Scleractinia	Cnidaria
8,531	Astrocoeniina	Cnidaria
7,513	Actiniaria	Cnidaria
7,432	Bolinopsidae	Ctenophora
7,273	Deuterostomia	—
6,961	Lophotrochozoa	—
5,998	Mollusca	Mollusca
5,611	Caenorhabditis	Nematoda

Supplementary Table 25: Top20 counts of orthologous group ancestors in BigWenDB. Columns indicate the number of orthologous groups specific for a given taxonomic group, its respective taxon, and the phylum to which this taxon belongs, if applicable. Numbers represent raw counts after determining group ancestors and may include false positives.

OG	Name	OG comp.	HMM hits	OG comp.	Ancestor	E-value
OG_5543	Evi1 (MECOM_HUMAN)	F 1; B 108	1. OG_5543	F 1; B 108	Bilateria	$0e^{-}$
			2. OG_7678	Cn 1; Ct 1; B 17	Metazoa	$1.6e^{-55}$
			3. OG_26723	25	Theria	$1.5e^{-54}$
			4. OG_2359	F 79; Pl 1; Po 6; Cn 9; Ct 8; B 95	Opisthokonta	$3.9e^{-54}$
OG_5226	odd-skipped related 1 (OSR1_HUMAN)	F 3; Cn 1; B 91	1. OG_5226	F 3; Cn 1; B 91	Opisthokonta	$1.4e^{-36}$
			2. OG_132128	Cn 1; B 5	Eumetazoa	$3.4e^{-28}$
			3. OG_59120	13	Protostomia	$1.5e^{-22}$
			4. OG_24005	Cn 5; B 8	Eumetazoa	$1.2e^{-19}$

Supplementary Table 26: HMM-HMM search results for kidney-related zinc finger transcription factors. Orthogroup ID, corresponding human gene name (UniProt ID), and orthogroup composition is shown for two bilaterian-specific zinc finger transcription factors related to kidney/nephron development. Columns 4–7 summarise results of HMM-HMM searches in our database. In column four and five, orthogroup ID and composition of the four best search hits for each query HMM are shown. Columns six and seven report their last common ancestor and corresponding E-value. B = Bilateria; Cn = Cnidaria; Ct = Ctenophora; F = Fungi; Pl = Placozoa; Po = Porifera. Note that each query HMM detects itself as best hit. In both cases, composition of the next similar orthogroups does not support a (eu)metazoan ancestor on a broad phylogenetic basis as only one or two non-bilaterian species are present in the detected orthogroups. This indicates that the two kidney-related factors originated in the bilaterian ancestor.

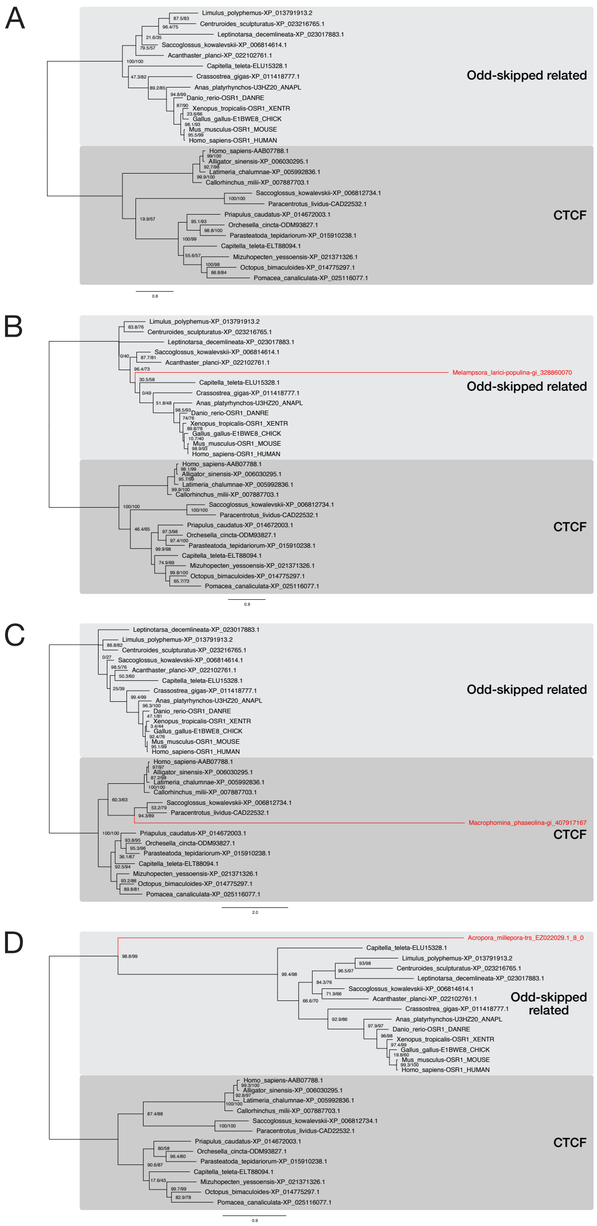

Supplementary Figure 1: Bilaterian-wide distribution of the Odd-skipped-related transcription factor. Multiple sequence alignments, underlying the four maximum likelihood phylogenetic reconstructions A–D, consist of 26 representative Odd-skipped related and CTCF sequences from various bilaterians, downloaded from NCBI. The alignments consist of 909–1094 distinct alignment patterns with a proportion of gaps and completely undetermined characters between 45.98 % and 52.38 %. In each subfigure B–D, one of three sequences (highlighted in red) from fungi or cnidarians was added that were present in the original orthogroup OG_5226 and shifted its ancestor to opisthokonts. The added sequences either reduce bootstrap support of the OSR clade (B) or clustered with the outgroup clade (C and D), suggesting that they do not belong to the OSR orthogroup. Branch labels correspond to the results of SH-aLRT (Shimodaira–Hasegawa-like approximate likelihood ratio test, left) and UFBoot (ultrafast bootstrap approximation, right) as implemented in IQ-TREE [Nguyen et al. 2015].

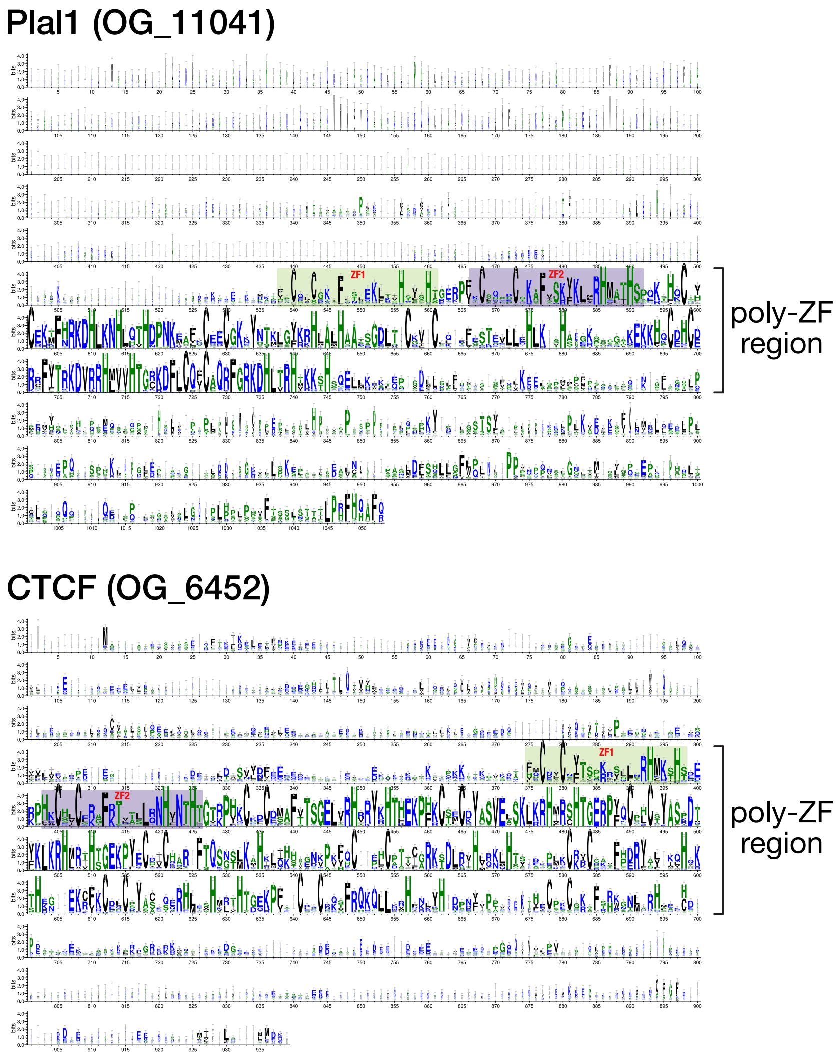

Supplementary Figure 2: Information content of two bilaterian-specific orthogroups with poly-zinc finger domain. Sequence logo representations [Crooks et al. 2004] of two bilaterian-specific orthogroups, CTCF (OG_6452) and Plal1 (OG_11041), both containing multiple C_2H_2 Zinc fingers in their central region (see Supplementary Table 8). The Plal1 logo was built from 92 sequences aligned over 1053 positions. The CTCF logo represents 175 sequences and 939 alignment positions, as obtained in the clustering. Indels and unaligned regions have been removed from the corresponding multiple sequence alignments, and the two first zinc finger domains of each protein are highlighted by shading and a red label. The sequence logos demonstrate that the central ZF domains are conserved beyond the Cys and His residues needed for Zn^{2+} complexion. Individual Zinc fingers in both proteins display highly informative and unique signatures that distinguish them from other Zinc fingers in the same or similar proteins.

References

Cande, J. D., Chopra, V. S., and Levine, M. (2009). Evolving enhancer-promoter interactions within the tinman complex of the flour beetle, Tribolium castaneum. *Development*, 136(18):3153–60.

Crooks, G. E., Hon, G., Chandonia, J.-M., and Brenner, S. E. (2004). Weblogo: a sequence logo generator. *Genome Research*, 14:1188–1190.

Heger, P., George, R., and Wiehe, T. (2013). Successive gain of insulator proteins in arthropod evolution. *Evolution*, 67(10):2945–2956.

Li, L., Stoeckert, Jr, C. J., and Roos, D. S. (2003). OrthoMCL: identification of ortholog groups for eukaryotic genomes. *Genome Research*, 13(9):2178–89.

Matus, D. Q., Thomsen, G. H., and Martindale, M. Q. (2007). FGF signaling in gastrulation and neural development in Nematostella vectensis, an anthozoan cnidarian. *Dev Genes Evol*, 217:137–148.

Nguyen, L.-T., Schmidt, H. A., von Haeseler, A., and Minh, B. Q. (2015). IQ-TREE: a fast and effective stochastic algorithm for estimating maximum-likelihood phylogenies. *Molecular Biology and Evolution*, 32:268–274.

Petryszak, R., Keays, M., Tang, Y. A., Fonseca, N. A., Barrera, E., Burdett, T., Füllgrabe, A., Fuentes, A. M.-P., Jupp, S., Koskinen, S., Mannion, O., Huerta, L., Megy, K., Snow, C., Williams, E., Barzine, M., Hastings, E., Weisser, H., Wright, J., Jaiswal, P., Huber, W., Choudhary, J., Parkinson, H. E., and Brazma, A. (2016). Expression atlas update—an integrated database of gene and protein expression in humans, animals and plants. *Nucleic Acids Res*, 44:D746–D752.

Sebé-Pedrós, A., Ariza-Cosano, A., Weirauch, M. T., Leininger, S., Yang, A., Torruella, G., Adamski, M., Adamska, M., Hughes, T. R., Gómez-Skarmeta, J. L., and Ruiz-Trillo, I. (2013). Early evolution of the T-box transcription factor family. *Proc Natl Acad Sci U S A*, 110:16050–16055.

Tomancak, P., Beaton, A., Weiszmann, R., Kwan, E., Shu, S., Lewis, S. E., Richards, S., Ashburner, M., Hartenstein, V., Celniker, S. E., and Rubin, G. M. (2002). Systematic determination of patterns of gene expression during Drosophila embryogenesis. *Genome Biol*, 3:RESEARCH0088.

Watanabe, H., Schmidt, H. A., Kuhn, A., Höger, S. K., Kocagöz, Y., Laumann-Lipp, N., Ozbek, S., and Holstein, T. W. (2014). Nodal signalling determines biradial asymmetry in Hydra. *Nature*, 515(7525):112–115.
