# Supplementary material for: The genetic factors of bilaterian evolution
Source: eLife. 2020 Jul 16;9:e45530. doi: 10.7554/eLife.45530 (PMC7535936; doi:10.7554/eLife.45530)
Supplement: Transparent reporting form [file elife-45530-transrepform.docx]

***eLife’s* transparent reporting form**

We encourage authors to provide detailed information *within their submission* to facilitate the interpretation and replication of experiments. Authors can upload supporting documentation to indicate the use of appropriate reporting guidelines for health-related research (see [EQUATOR Network](http://www.equator-network.org/%20)), life science research (see the [BioSharing Information Resource](https://biosharing.org/)), or the [ARRIVE guidelines](http://www.plosbiology.org/article/info:doi/10.1371/journal.pbio.1000412) for reporting work involving animal research. Where applicable, authors should refer to any relevant reporting standards documents in this form.

If you have any questions, please consult our Journal Policies and/or contact us: [editorial@elifesciences.org](mailto:editorial@elifesciences.org).

**Sample-size estimation**

- You should state whether an appropriate sample size was computed when the study was being designed
- You should state the statistical method of sample size computation and any required assumptions
- If no explicit power analysis was used, you should describe how you decided what sample (replicate) size (number) to use

Please outline where this information can be found within the submission (e.g., sections or figure legends), or explain why this information doesn’t apply to your submission:

No statistical methods were used to determine appropriate sample sizes. To identify bilaterian-specific orthologue groups, we collected a dataset as representative and comprehensive as possible while maintaining computational feasibility. To achieve good resolution at the base of bilaterians, we included all sequence data from non-bilaterian animals available at the time. The dataset is described in Figure 1 – figure supplement 1; Supplementary files 1 and 2.

**Replicates**

- You should report how often each experiment was performed
- You should include a definition of biological versus technical replication
- The data obtained should be provided and sufficient information should be provided to indicate the number of independent biological and/or technical replicates
- If you encountered any outliers, you should describe how these were handled
- Criteria for exclusion/inclusion of data should be clearly stated
- High-throughput sequence data should be uploaded before submission, with a private link for reviewers provided (these are available from both GEO and ArrayExpress)

Please outline where this information can be found within the submission (e.g., sections or figure legends), or explain why this information doesn’t apply to your submission:

Not applicable since this study describes a bioinformatics pipeline processing previously published datasets.

**Statistical reporting**

- Statistical analysis methods should be described and justified
- Raw data should be presented in figures whenever informative to do so (typically when N per group is less than 10)
- For each experiment, you should identify the statistical tests used, exact values of N, definitions of center, methods of multiple test correction, and dispersion and precision measures (e.g., mean, median, SD, SEM, confidence intervals; and, for the major substantive results, a measure of effect size (e.g., Pearson's r, Cohen's d)
- Report exact p-values wherever possible alongside the summary statistics and 95% confidence intervals. These should be reported for all key questions and not only when the p-value is less than 0.05.

Please outline where this information can be found within the submission (e.g., sections or figure legends), or explain why this information doesn’t apply to your submission:

We have not used statistical significance analysis for the main results of the study (orthology clustering). However, we investigated particular aspects of the study using statistical methods. In Figure 8, for example, we show slightly more protein-protein interactions among bilaterian-specific proteins than among older proteins. The relevant tests are mentioned on p16 main text and p25 in Material and methods. In Figure 2 – figure supplement 1, we show the enrichment of transcription factors in the bilaterian-specific gene set, with the corresponding statistical tests mentioned on p5 main text and p23 Material and methods.

(For large datasets, or papers with a very large number of statistical tests, you may upload a single table file with tests, Ns, etc., with reference to sections in the manuscript.)

**Group allocation**

- Indicate how samples were allocated into experimental groups (in the case of clinical studies, please specify allocation to treatment method); if randomization was used, please also state if restricted randomization was applied
- Indicate if masking was used during group allocation, data collection and/or data analysis

Please outline where this information can be found within the submission (e.g., sections or figure legends), or explain why this information doesn’t apply to your submission:

Not applicable since our experiments constitute computational simulations. Sequence samples were allocated to the groups Bilateria and non-Bilateria on the basis of their accepted taxonomy (NCBI taxonomy; ftp://ftp.ncbi.nlm. nih.gov/pub/taxonomy/taxdump.tar.gz). Bilaterian-specific orthologue groups were selected as described on p39 (Material and methods).

**Additional data files (“source data”)**

- We encourage you to upload relevant additional data files, such as numerical data that are represented as a graph in a figure, or as a summary table
- Where provided, these should be in the most useful format, and they can be uploaded as “Source data” files linked to a main figure or table
- Include model definition files including the full list of parameters used
- Include code used for data analysis (e.g., R, MatLab)
- Avoid stating that data files are “available upon request”

Please indicate the figures or tables for which source data files have been provided:

The sequence sources used for this study are described in Figure 1 – figure supplement 1 and Supplementary files 1 and 2; the R code developed for orthology clustering is documented at https://github.com/prheger/BigWenDB; clustering results as well as the sequence set used to obtain them are available at https://datadryad.org/stash/dataset/doi:10.5061/dryad.4qf7168.
